# The Influence of Back Pain and Urinary Incontinence on Daily Tasks of Mothers at 12 Months Postpartum

**DOI:** 10.1371/journal.pone.0129615

**Published:** 2015-06-17

**Authors:** Cynthia A. Mannion, Angela E. Vinturache, Sheila W. McDonald, Suzanne C. Tough

**Affiliations:** 1 Faculty of Nursing, University of Calgary, Calgary, Alberta, Canada; 2 Department of Paediatrics, Faculty of Medicine, University of Calgary, Calgary, Alberta, Canada; 3 Department of Community Health Sciences, Faculty of Medicine, University of Calgary, Calgary, Alberta, Canada; Hunter College, UNITED STATES

## Abstract

**Objective:**

The present study examined back pain (BP) and/or urinary incontinence (UI) impact on the ability to perform daily tasks at 12 months after childbirth in healthy reproductive women who sought maternity care in community based family practice clinics.

**Methods:**

This study is a secondary analysis from the All Our Babies Study, a prospective, community-based pregnancy cohort in Calgary, Alberta. Maternal self-reported information on demographics, lifestyle, experiences with pregnancy and childbirth, occurrence of BP, UI and consequent impairment of daily tasks were collected by questionnaires administered before 25 weeks, at 34-36 weeks gestation and at 4 and 12 months postpartum. The occurrence and severity of BP and UI at one year after childbirth was assessed using descriptive and bivariate analyses. Logistic regression models examined the association between demographic and obstetrical variables and the severity of functional impairment due to UI and BP.

**Results:**

From 1574 women with singleton pregnancies included in the study, 1212 (77%) experienced BP, 773 (49%) UI, and 620 (40%) both BP and UI. From the 821 women reporting impairment of daily tasks due to BP, 199 (24 %) were moderately and 90 (11%) severely affected with the remainder, 532 (64%) being mildly affected. From 267 women with functional impairment due to UI, 52 (19%) reported moderately to severe impairment in their ability to perform daily tasks. Obesity and parity were risk factors for impairment of daily functioning due to BP, whereas obesity and vaginal delivery increased the risk of moderate to severe impairment due to UI.

**Conclusions:**

BP and UI are common occurrences 1 year after childbirth. Maternal performance of daily tasks and women’s health and quality of life are more often impaired due to BP than UI. Our study brings new evidence of the risk factors that predict severity and impact of these conditions on women functioning at 12 months postpartum.

## Introduction

Current evidence indicates that urinary incontinence (UI) and back pain (BP) following pregnancy and childbirth are associated with functional impairment and lifestyle alterations for postpartum women. In addition, UI is embarrassing, debilitating, and threatens women’s self-esteem, body image, and sexual activities [1]. The number of women reporting these afflictions raises quality of life issues and medical concerns associated with childbirth.

The prevalence of UI varies widely with reports from 6 to 43% [2] and most recently to affect 18.6 to 60% of postpartum women [3]. Similarly, the prevalence of BP in postpartum has been reported between 3.9 and 89.9% [4]. The large variations in prevalence can be attributed to choice of study designs, heterogeneity of samples, variability of assessment tools, the time interval after childbirth when assessed as well as clinical differences in the management of labour and delivery. Some women may experience both BP and UI but there are limited data on the prevalence of both conditions and their combined effect on quality of life [5]. Recent studies suggest a relationship between UI and BP in general population. Women with pre-existing UI had increased risk for development of BP, and conversely, women with pre-existing BP were more likely to develop UI [6–8]. A similar relationship between UI and BP was observed during pregnancy, pregnant women who had UI having increased odds for reporting coexistent BP [9]. However, the relationship between UI and BP in postpartum is understudied and underreported.

There are a number of explanatory factors contributing to UI in reproductive women. In a systematic review, Wohlrab and Rardin (2008) demonstrated that UI may be related to pregnancy independent from mode of delivery be it spontaneous vaginal, assisted delivery (forceps, vacuum extraction) or caesarean section (C-section)[1]. However, it is difficult to assess UI resulting exclusively from pregnancy, as UI was also reported in nulliparous women with prevalence ranging from 8% to 32% [10].

Several large epidemiologic studies have reported on the relationship between UI and mode of delivery [11, 12]. Vaginal delivery has been shown to be an independent risk factor for the persistence of UI in the first three postpartum months [13]. Several other studies support increased risk of UI with vaginal delivery and imply that C-section may be protective of UI [1, 11, 14–16]. Obstetrical management of labour and delivery resulting in trauma, severe tearing and episiotomy have been reported to contribute to development of UI after childbirth [14]. Other UI predictors included age at delivery, parity, obesity, maternal smoking and infant birth weight > 4000 g [17].

BP is one of the most frequently reported complaints during pregnancy [18], and postpartum period [4, 19, 20]. Many women enter pregnancy with a previous history of BP possibly associated with occupational hazards or a chronic health condition [21, 22]. The reported prevalence of BP at one year postpartum ranged from 33.7% to 64.7% [21]. In a prospective cohort of low obstetrical risk women, from 76% of women experienced BP during pregnancy 21% had persistent BP at 2 years postpartum [22]. Several risk factors including parity, age, body mass index (BMI), infant birth weight, mode of delivery and the use of epidural analgesia, have been implicated in the frequency and severity of BP symptoms but the results among studies are conflicting and inconclusive [23] [24].

The impact of BP and UI upon maternal performance of daily tasks during the postpartum period is largely unstudied [9, 25, 26]. Ostgaard *et al* [27] and Noren *et al* [28] reported that BP compromises maternal ability to work during pregnancy and interferes with activities of daily living (ADL). Wang *et al* (2004) concur, showing that approximately half of the women with moderate to severe BP experience impaired performance of daily tasks during pregnancy [25]. It is likely that the effect of BP and/or UI on maternal performance of daily tasks is related to the severity of each or both conditions. Therefore, we anticipated that at 12 months postpartum, some women would experience BP and/or UI, and the severity of these conditions could directly influence maternal performance of daily tasks. The purpose of this study was to describe the prevalence and severity of self-reported BP and UI in the first postpartum year in a cohort of women who received prenatal care in community health care centers. In addition, we assessed the relationship between the self-reported severity of symptoms and maternal performance of daily tasks at 12 months postpartum.

## Methods

### Study population

This study is a secondary analysis of The All Our Babies Study, a prospective community-based pregnancy cohort studied in Calgary, Alberta (n = 3388). Information about the recruitment, eligibility, and data collection for the cohort has been described in detail in a previous publication (McDonald *et al* 2013). Participants were recruited during their first trimester of pregnancy between May 2008 and December 2010 and completed questionnaires prior to 25 weeks, between 34–36 weeks gestation, and at approximately 4 and 12 months postpartum. Information was collected on demographics, lifestyle, mental, psychosocial and physical health, pregnancy history, health service utilization, quality of life, and breastfeeding. Surveys were linked to electronic health records at hospital admissions for labour and delivery using unique identifiers. The medical records provided additional and pertinent details on pregnancy complications and birth outcomes. Questions about the presence and severity of BP and UI and performance of daily tasks were asked at 12 months postpartum. For the purposes of this study, women who delivered singletons, who were 12 months old at the time of survey distribution were eligible to complete the questionnaire and were included in the study (n = 1574).

The All Our Babies Study was approved by the Child Health Research Office, Alberta Children’s Hospital, Alberta Health Services, and the Conjoint Health Research Ethics Board of the Faculties of Medicine, University of Calgary. Written informed consent was obtained from the study participants at the time of recruitment, who were also provided copies for their records.

### Data Collection

Data was collected on participants’ socio-demographics (maternal age, marital status, income, education, ethnicity), pre-pregnancy BMI (underweight, normal weight, overweight, obese), onset of labour (spontaneous or induction), delivery mode (spontaneous vaginal, assisted vaginal (vacuum and forceps), and emergency or elective C-section), obstetrical analgesia (epidural, spinal, no analgesia), parity (primiparous, multiparous), gravidity (primigravida, multigravida), birth weight, and pregnancy complications (gestational diabetes, pregnancy induced hypertension, preeclampsia and placenta previa). Initial questions polled the presence and frequency of BP and UI with the following questions: “Since your baby’s birth, have you experienced back pain?” and “Since your baby’s birth have you experienced urinary incontinence (unintentional loss of urine?). The answers were recorded on a 5-item scale (yes, all of the time; yes, most of the time; yes, some of the time; once or twice, and no), that captured the frequency of BP, and the unintentional loss of urine.

Women who answered yes to initial questions were categorized as having BP and/or UI. Severity of BP was categorised on the self-reported frequency of symptoms: mild (some of the time or once or twice), moderate (most of the time), and severe (all of the time). Severity of UI was similarly categorised on the frequency of leakage of urine: mild (some of the time or once or twice), moderate (most of the time), and severe (all of the time). Due to low cell count for reports on moderate and severe UI, the last two categories were combined into one category moderate/severe (most or all of the time). These participants were also asked to evaluate the level of functional impairment of daily tasks caused by either BP or UI on a Likert scale. Maternal performance of daily tasks included both maternal tasks (i.e. caring for the baby) and routine household tasks (i.e. performing household chores). The effect of BP on daily tasks was graded on a scale anchored by 1 (has not affected my ability) to 10 (has, at times, prevented me from doing any of my daily tasks) and categorized as mild (2–4 rating), moderate (5–7 rating) and severe (8–10 rating) impairment in performance of daily tasks. The effect of UI on tasks was assessed using a similar scale. Due to low cell count for reports on moderate and severe functional impairment due to UI, the last two categories were combined in one category, moderate/severe (5–10 rating). The questionnaire did not include items to allow identification of the anatomical site (i.e. thoracic, scapular, lumbar or pelvic girdle pain), intensity or duration of the BP symptoms. Also, participants were not asked specific questions for identification of the type of UI (i.e. urge, stress or mixed) or intensity of urine leakage.

BMI [weight (kg)/height (m)^2^] was calculated on self-reported height and weight prior to pregnancy. Using the World Health Organization’s BMI classification (2000), women were categorized as underweight (BMI<18.50 kg/m^2^), normal weight (BMI 18.50–24.99 kg/m^2^), overweight (BMI 25.00–29.99 kg/m^2^), and obese (BMI >30.00 kg/m^2^) [29].

### Data Analysis

Descriptive statistics were used to describe all study variables. Means, standard deviations, and percentages were used to summarize continuous data, and frequency distributions were used to summarize categorical data. The Chi-square and Fisher’s exact test examined the associations between presence and severity of BP and UI, maternal performance of daily tasks and socio- demographic and obstetrical variables.

Because the level of impairment of daily tasks due to BP had three levels (mild, moderate and severe), a multinomial regression analysis was performed to identify potential predictors of severity of impaired maternal performance of daily tasks attributed to BP at 12 months postpartum using demographic and obstetrical variables and controlling for any confounding influence of UI symptoms. Multivariable logistic regression was performed to establish the obstetrical and socio-demographic variables predictive of severity of functional impairment due to UI at 12 months postpartum (the variable ‘severity of impairment of daily tasks due to UI’ had two levels, mild and moderate/severe). This model also controlled for BP as a potential confounder for the degree of impairment caused by co-occurrence of UI. Independent variables in the models for both BP and UI included maternal age [25, 26], BMI [30], parity, gestational age (GA), infant birth weight [26], mode of delivery, and pregnancy complications [18]. The confounders were included a priori in the analysis based on their clinical relevance and previous reports from literature [18], recognizing the potential for over-adjustment when controlling for these variables, and, therefore, possible underestimation of odds ratios for some predictors, given that colliders and mediators, depending of the predictor, may be present.

Regression models were performed using all women, not only those who indicated that they had experienced the condition (back pain and/or urinary incontinence). Women who did not experience the condition were collapsed in the category that also comprised women who did experience the condition but who indicated low severity or mild impairment. Odds ratios (ORs) were calculated with 95% confidence intervals (CI) and all associations were considered statistically significant at p<0.05. SPSS version 20 (IBM SPSS, Chicago, IL) was used for analyses.

## Results

### Population demographic and clinical characteristics

Table 1 summarizes the characteristics of the women included in the study. The majority of the women included were Caucasian, had lived in Canada more than 5 years, were married or in a common law relationship, had attained postsecondary or higher level of education, and had a household income >$80,000. These socio-demographic characteristics of our sample align with the pregnant and parenting population of an urban centre in Western Canada and reflect the population living in Calgary and area [31].

**Table 1:** Characteristics of the study participants (N = 1574).

Characteristics	n (%)
**Maternal age in years** *Mean (SD)*	31.45 (4.43)
**Education** ^**1**^	
High School	360 (23.0)
Postsecondary	1207 (77.0)
**Household income**	
≥$80,000	1084 (71.6)
<$80,000	431 (28.4)
**Marital status**	
Married/Common-law	1498 (95.5)
Single	70 (4.5)
**Ethnicity**	
White/Caucasian	1292 (82.4)
Other	276 (17.6)
**Time in Canada**	
Born in Canada/Lived in Canada for ≥5 years	1424 (91.1)
Lived in Canada for less than 5 years	139 (8.9)
**Gravidity**	
Primigravida	548 (35.1)
Multigravida	1012 (64.9)
**Parity**	
Primiparous	681 (47.7)
Multiparous	747 (52.3)
**Pre-pregnancy BMI**	
Underweight (BMI< 18.50 kg/m^2^)	54 (3.5)
Normal weight (BMI 18.50–24.99 kg/m^2^)	937 (60.9)
Overweight (BMI 25.00–29.99 kg/m^2^)	343 (22.3)
Obese/Morbidly obese (BMI ≥30.00 kg/m^2^)	204 (13.3)
**Pregnancy complications** ^**2**^	
Yes	201 (14.1)
No	1228 (85.9)
**Onset of labour**	
Spontaneous onset	971 (72.1)
Induction of labour	376 (27.9)
**Mode of delivery**	
Spontaneous vaginal	1013 (70.9)
Assisted vaginal delivery (vacuum and/or forceps)	36 (2.5)
Emergency or elective caesarean section	380 (26.6)
**Obstetrical analgesia**	
Epidural/spinal	743 (52.0)
No analgesia	686 (48.0)
**Gestational age** (weeks), *mean (SD)*	38.86 (1.89)
**Birth weight** (grams), *mean (SD)*	3357.5 (547.7)
**Breastfeeding status**	
Did not breastfeed	60 (3.8)
Breastfed <24 weeks	339 (21.6)
Breastfed ≥24 weeks	1173 (74.6)
**Back pain at 12 months postpartum**	1212 (77.0)
**Back pain affected ability to conduct daily tasks on a scale of 0 to 10**	
No back pain or not affected (0–1)	750 (47.7)
Mildly affected (2–4)	532 (33.9)
Moderately affected (5–7)	199 (12.7)
Severely affected (8–10)	90 (5.7)
**Urinary incontinence at 12 months postpartum**	773 (49.2)
**Urinary incontinence affected ability to conduct daily tasks on a scale of 0 to 10**	
No urinary incontinence or not affected (0–1)	1305 (83.0)
Mildly affected (2–4)	215 (13.7)
Moderately affected (5–7)	40 (2.5)
Severely affected (8–10)	12 (0.8)

^1^The education categories included: for the High school category: some high school, graduated high school, some post-secondary, and for the Post-secondary category: graduated post-secondary, some graduate school, or completed graduate school

^2^Pregnancy complications included at least one of the following: pregnancy induced hypertension, pre-eclampsia, eclampsia, placental abruption, gestational diabetes, or placenta praevia)

The sample from our study is representative of the urban parenting population in Canada. In comparison with Calgary, Alberta and Canadian statistics the characteristics of women recruited into the AOB study show very similar demographics across categories (maternal age, place of birth, ethnicity, education, marital status, and parity), with some exceptions such as income levels. A high level of agreement was found between perinatal indicators (preterm birth, intrauterine growth restriction rates) from the AOB study and provincial and national rates [31, 32]. In depth details regarding the representativeness of AOB sample are presented in McDonald *et al* [31].

The mean age of participants at the time of the study was 31.5 (± 4.4 SD, range 18–43) years. Based on pre-pregnancy BMI, 60.9% were normal weight, with the rest being overweight (22.3%), obese (13.3%), and a small percentage (3.5%) of all participants were underweight. The majority of women were multigravida (65%). Primiparous women made up 48% (n = 747) of the sample. Almost 200 women (14%) experienced pregnancy complications such as pregnancy induced hypertensive disorder, gestational diabetes, and placenta praevia. Just over one thousand women (70.9%) had a spontaneous vaginal delivery; 28.6% (n = 380) required obstetrical management including C-section and 2.5% (n = 36) had an assisted vaginal delivery. Mean GA at delivery was 38.9 weeks (±1.9SD, range 23–42 weeks), with a preterm birth (GA at delivery ≤ 36^6/7^ weeks) rate of 6.5% (93 infants). Mean birth weight was 3357.5 g (±547.6 SD, range 595–5071 g).

### Back pain and impact on daily tasks at 12 months postpartum

During twelve months postpartum, 77% (n = 1212) of study participants reported some level of BP (Table 1). No differences were observed for ethnicity, household income, level of education, and marital status between women who reported BP and those who did not (Table 2). However, women who were obese prior to pregnancy reported BP more frequently at 12 months postpartum (p = 0.018) than normal or underweight women. In addition, there were no differences between obstetrical characteristics of women who experienced BP and those who did not considering gravidity, parity, and mode of delivery or infants’ GA at delivery. Women who had induction of labour were more likely to report BP at 12 months postpartum (p = 0.015) than women who experienced spontaneous onset of labour.

**Table 2:** Severity of symptoms and effect on maternal performance of daily tasks due to back pain at 12 months postpartum.

Variables^1^	Back pain			Severity of symptoms of back pain				Effects of back pain on performance of daily tasks			
	BP	No BP	p-values^2^	Mild	Moderate	Severe	p-values^3^	Mild	Moderate	Severe	p-values^4^
**Demographics**											
**Maternal age at delivery** (years)	31.3 (4.5)	31.8 (4.1)	0.124	31.5 (4.4)	31.0 (4.7)	30.5 (4.7)	0.192	31.1 (4.5)	31.3 (4.2)	31.6 (4.7)	0.683
**Ethnicity**											
White/Caucasian	983 (81.4)	308 (85.8)	0.057	805 (82.9)	144 (81.4)	34 (58.6)	**≤0.001**	438 (82.6)	148 (75.5)	75 (83.3)	0.079
Other	224 (18.6)	51 (14.2)		166 (17.1)	33 (18.6)	24 (41.4)		92 (17.4)	48 (24.5)	15 (16.7)	
**Household income**											
< $80,000	345 (29.6)	85 (24.6)	0.07	252 (26.8)	65 (38.0)	28 (50.9)	≤0.001	165 (32.0)	68 (35.8)	33 (39.8)	0.306
≥ $80,000	822 (70.4)	261 (75.4)		688 (73.2)	106 (62.0)	27 (49.1)		350 (68.0)	122 (64.2)	50 (60.2)	
**Education** ^**5**^											
High school	288 (23.9)	72 (20.1)	0.139	227 (23.4)	42 (23.9)	18 (31.0)	0.41	123 (23.3)	46 (23.4)	27 (30.0)	0.371
Post-secondary and higher	919 (76.1)	286 (79.9)		745 (76.6)	134 (76.1)	40 (69.0)		406 (76.7)	151 (76.6)	63 (70.0)	
**Marital Status**											
Married/Common law	1150 (95.3)	347 (96.7)	0.263	928 (95.7)	167 (93.8)	54 (93.1)	0.411	507 (95.7)	186 (94.4)	86 (95.6)	0.775
Single	57 (4.7)	12 (3.3)		42 (4.3)	11 (6.2)	4 (6.9)		23 (4.3)	11 (5.6)	4 (4.4)	
**Pre-pregnancy body mass index** (BMI, kg/m^2^)											
Underweight (< 18.50)	44 (3.7)	10 (2.9)	0.018	35 (3.7)	6 (3.4)	3 (5.4)	0.164	15 (2.9)	8 (4.2)	2 (2.2)	0.251
Normal weight (18.50–24.99)	712 (60.0)	223 (63.7)		579 (60.6)	97 (55.7)	35 (62.5)		324 (62.0)	103 (54.2)	47 (52.8)	
Overweight (25.00–29.99)	256 (21.6)	87 (24.9)		211 (22.1)	33 (19.0)	12 (21.4)		110 (21.0)	41 (21.6)	21 (23.6)	
Obese (≥30.00)	174 (14.7)	30 (8.6)		130 (13.6)	38 (21.8)	6 (10.7)		74 (14.1)	38 (20.0)	19 (21.3)	
**Obstetrical characteristics**											
**Gravidity**											
Primigravida	414 (34.5)	134 (37.5)	0.287	331 (34.3)	63 (35.4)	19 (32.8)	0.928	184 (35.0)	66 (33.5)	26 (28.9)	0.523
Multigravida	787 (65.5)	223 (62.5)		633 (65.7)	115 (64.6)	39 (67.2)		342 (65.0)	131 (66.5)	64 (71.1)	
**Parity**											
Primipara	522 (47.4)	157 (48.5)	0.73	419 (47.5)	77 (47.2)	26 (45.6)	0.962	234 (49.0)	81 (44.8)	30 (36.6)	0.099
Multipara	580 (52.6)	167 (51.5)		463 (52.5)	86 (52.8)	31 (54.4)		244 (51.0)	100 (55.2)	52 (63.4)	
**Type of labour onset**											
Induced labour	307 (29.5)	68 (22.4)	0.015	255 (30.5)	40 (26.1)	12 (22.6)	0.292	134 (29.3)	49 (28.8)	23 (30.3)	0.974
Spontaneous labour	734 (70.5)	236 (77.6)		580 (69.5)	113 (73.9)	41 (77.4)		323 (70.7)	121 (71.2)	53 (69.7)	
**Mode of delivery** ^**6**^											
Spontaneous vaginal	784 (71.1)	229 (70.7)	0.11	638 (72.3)	113 (69.3)	33 (57.9)	0.072	336 (70.3)	130 (71.8)	57 (69.5)	0.406
Assisted vaginal delivery	22 (2.0)	13 (4.0)		19 (2.2)	3 (1.8)	0 (0.0)		9 (1.9)	6 (3.3)	0 (0.0)	
Caesarean section	297 (26.9)	82 (25.3)		226 (25.6)	47 (28.8)	24 (42.1)		133 (27.8)	45 (24.9)	25 (30.5)	
**Obstetrical analgesia**											
Epidural/spinal	578 (52.4)	164 (50.6)	0.572	466 (52.8)	84 (51.5)	28 (49.1)	0.842	260 (54.4)	94 (51.9)	38 (46.3)	0.385
No analgesia	525 (47.6)	160 (49.4)		417 (47.2)	79 (48.5)	29 (50.9)		218 (45.6)	87 (48.1)	44 (53.7)	
**Preterm birth prevalence**											
GA< 37 weeks (preterm)	72 (6.5)	21 (6.5)	0.976	56 (6.3)	9 (5.5)	7 (12.3)	0.182	31 (6.5)	12 (6.6)	11 (13.4)	0.077
GA ≥ 37 weeks (term)	1031 (93.5)	303 (93.5)		827 (93.7)	154 (94.5)	50 (87.7)		447 (93.5)	169 (93.4)	71 (86.6)	
**Gestational age** (weeks)	38.8 (1.8)	38.8 (1.9)	0.993	38.9 (1.8)	38.7 (1.7)	38.8 (1.9)	0.568	38.8 (1.9)	38.8 (1.7)	38.4 (2.3)	0.213
**Birth weight** (grams)	3368.9	3323.1	0.916	3380.1	3319.1	3337.8	0.385	3351.6	3362.7	3338.6	0.946
	(546.5)	(549.4)		(546.0)	(529.7)	(599.7)		(548.9)	(534.6)	(672.2)	

^1^Variables are presented as n (%), except for maternal age, gestational age at delivery and infant birth weight which are presented as mean and (SD)

^**2**^ Comparisons between women who did and who did not experience back pain at 12 months postpartum

^3^ Comparisons between women with mild, moderate and severe symptoms of back pain

^4^ Comparisons between women who reported mild, moderate and severe impairment of daily tasks due to back pain

^5^The education categories included: for the High school category: some high school, graduated high school, or some post-secondary, and for Post-secondary category: graduated post-secondary, some graduate school, or completed graduate school

^6^Assisted vaginal deliveries included forceps, vacuum or both forceps and vacuum; Caesarean section included both emergency and elective caesarean sections

*p-values* from Chi square tests

Percentages are calculated per column for each variable

In women who experienced BP, the severity of symptoms reported at 12 months after childbirth was influenced by demographic characteristics and was not affected by the obstetrical history or the events at labour and delivery. For example, women with lower household income levels and who were ethnicities other than Caucasian were more likely to report severe back pain (p ≤ 0.001 for both) (Table 2).

Although some level of BP was experienced by the majority of women at 12 months postpartum, there were divided effects on maternal performance of daily tasks. More than half, 67.7% (n = 821) reported various detrimental effects on maternal performance of daily tasks. Almost 64% (n = 532) from the women with functional impairment due to BP were mildly affected, 24% (n = 199) were moderately affected, and 11% (n = 90) women reported BP severely impeded their ability to perform daily tasks (Tables 1 and 2). A significant association was observed between frequency of BP symptoms and impairment of daily tasks (p<0.001).

There were no differences in clinical or demographic characteristics between women who experienced mild impairment compared to women who experienced moderate to severe impairment of daily tasks due to BP (Table 2).

In multinomial analysis (Table 3), compared to women without or with mild functional impairment due to BP, women who were obese were at increased risk for moderate BP (adjusted OR 1.70, CI 1.07, 2.68), and women who were multiparous were at increased risk of severe impairment of daily tasks due to BP (adjusted OR 1.80, CI 1.10, 2.95). UI was not a contributory factor to the severity of functional impairment induced by BP at one year after childbirth.

**Table 3:** Crude and adjusted odds ratios and 95% confidence intervals for factor related to severity of impairment on daily tasks due to back pain and urinary incontinence at 12 months postpartum.

	Moderate impairment of daily tasks due to backpain^1^		Severe impairment of daily tasks due to backpain^1^		Moderate/severe impairment of daily tasks due to UI^1^	
	uOR (95% CI)	aOR (95% CI)	uOR (95% CI)	aOR (95% CI)	uOR (95% CI)	aOR (95% CI)
Maternal age^2^ (years)	0.99 (0.96, 1.03)	0.99 (0.95, 1.03)	1.00 (0.96, 1.06)	0.99 (0.94, 1.05)	**1.10 (1.03, 1.17)**	1.10 (1.03, 1.18*)
Pre-pregnancy BMI (kg/m^2^)						
Normal weight (BMI 18.5 —-24.99)^3^	1.0	1.0	1.0	1.0	1.0	1.0
Overweight (BMI 25.00–29.99)	1.11 (0.75, 1.64)	1.04 (0.69, 1.57)	1.25 (0.73, 2.12)	0.97 (0.54, 1.75)	1.38 (0.68, 2.78)	1.34 (0.64, 2.79)
Obese (BMI ≥30.00)	**1.97 (1.30, 2.97)**	1.70 (1.07, 2.68*)	**2.16 (1.23, 3.78)**	1.59 (0.85, 2.97)	**3.01 (1.55, 5.85)**	2.84 (1.34, 6.02*)
Parity						
Primiparity^3^	1.0	1.0	1.0	1.0	1.0	1.0
Multiparity	1.18 (0.86, 1.61)	1.24 (0.88, 1.75)	**1.65 (1.04, 2.63)**	**1.80 (1.10,2.95)**	1.40 (0.78, 2.53)	1.29 (0.70, 2.37)
Gestational age^2^ (weeks)	0.97 (0.90, 1.06)	1.03 (0.93, 1.13)	**0.90 (0.82, 0.99)**	0.91 (0.82, 1.01)	0.99 (0.85, 1.14)	1.05 (0.88, 1.25)
Birth weight						
Birth weight > 4,000 g (macrosoms)^3^	1.0	1.0	1.0	1.0	1.0	1.0
Birth weight ≤3,999 g (normosoms)	0.98 (0.59, 1.61)	1.02 (0.60, 1.74)	1.12 (0.53, 2.39)	1.12 (0.52, 2.45)	2.85 (0.69, 11.86)	3.45 (0.81, 14.77)
Mode of delivery						
Cesarean section^3^	1.0	1.0	1.0	1.0	1.0	1.0
Vaginal delivery (spontaneous or operative (vacuum and/or forceps)	1.09 (0.76, 1.56)	1.27 (0.84, 1.91)	0.82 (0.50, 1.34)	0.86 (0.51, 1.45)	**3.20 (1.26, 8.14)**	**3.94 (1.52, 10.23)**
Pregnancy complications^4^						
No^3^	1.0	1.0	1.0	1.0	1.0	1.0
Yes	**1.63 (1.09, 2.45)**	1.51 (0.95, 2.40)	1.65 (0.93, 2.93)	1.27 (0.67, 2.40)	**2.37 (1.23, 4.56)**	1.97 (0.94, 4.13)
Urinary incontinence since giving birth					-	-
No^3^	1.0	1.0	1.0	1.0		
Yes	1.27 (0.94, 1.71)	1.23 (0.87, 1.72)	1.49 (0.97, 2.30)	1.38 (0.85, 2.23)		
Back pain since giving birth	-	-	-	-		
No^3^					1.0	1.0
Yes					1.66 (0.77, 3.55)	1.50 (0.68, 3.30)

^1^Reference category: no or mild functional impairment of daily tasks due to back pain or urinary incontinence, respectively

^2^Continuous variables

^3^Reference category

^4^Complications included pregnancy-induced hypertension, preeclampsia, gestational diabetes, placenta praevia

uOR, unadjusted odds ratios; aOR, adjusted odds ratios; CI, confidence intervals

Odds rations and confidence intervals are adjusted for all variables in this table

### Urinary incontinence and impact on daily tasks at 12 months postpartum

As shown in Table 4, there was no difference between women who reported UI and women who did not with regard to education, marital status and body weight. Women who reported symptoms of UI 12 months postpartum were more frequently Caucasian (p<0.001) and had higher household income than $80,000 (p = 0.036). Women who delivered vaginally were at elevated risk for UI (p<0.05). There was no association between UI in postpartum and the number of previous pregnancies (p = 0.08) and deliveries (p = 0.10), obstetrical analgesia (p = 0.07) or type of labour onset (p = 0.24).

**Table 4:** Severity of symptoms and level of impairment of daily tasks due to urinary incontinence at 12 months post-partum.

Variables^1^	Urinary Incontinence			Severity of urinary incontinence symptoms			Impairment of daily tasks due to urinary incontinence		
	UI	No UI	p-values^2^	Mild	Moderate/Severe	p-values^3^	Mild	Moderate/ Severe	p-values^4^
**Demographics**									
**Maternal age at delivery (years)**	31.7 (4.5)	31.2 (4.3)	0.022	31.6 (4.5)	33.7 (4.6)	0.004	31.9 (4.6)	33.2 (4.0)	0.088
**Ethnicity**									
White/Caucasian	663 (85.9)	628 (79.1)	<0.001	626 (85.9)	37 (86.0)	0.974	183 (85.1)	41 (80.4)	0.406
Other	109 (14.1)	166 (20.9)		103 (14.1)	6 (14.0)		32 (14.9)	10 (19.6)	
**Household income**									
< $80,000	195 (26.0)	235 (30.8)	0.036	182 (25.6)	13 (32.5)	0.333	57 (27.1)	13 (27.7)	0.943
≥ $80,000	556 (74.0)	527 (69.2)		529 (74.4)	27 (67.5)		153 (72.9)	34 (72.3)	
**Education** ^5^									
High school	172 (22.3)	188 (23.7)	0.502	157 (21.5)	15 (34.9)	0.041	42 (19.5)	14 (27.5)	0.213
Post-secondary	600 (77.7)	605 (76.3)		572 (78.5)	28 (65.1)		173 (80.5)	37 (72.5)	
**Marital status**									
Married/Common law	733 (94.9)	764 (96.2)	0.22	695 (95.3)	38 (88.4)	0.059	201 (93.5)	49 (94.2)	1
Single	39 (5.1)	30 (3.8)		34 (4.7)	5 (11.6)		14 (6.5)	3 (5.8)	
**Pre-pregnancy body mass index** (BMI, kg/m^2^)									
Underweight (< 18.50)	22 (2.9)	32 (4.2)	0.152	22 (3.0)	0 (0.0)	<0.001	2 (0.9)	1 (1.9)	0.225
Normal weight (18.50–24.99)	452 (59.1)	483 (62.6)		437 (60.5)	15 (34.9)		121 (57.1)	24 (46.2)	
Overweight (25.00–29.99)	180 (23.5)	163 (21.1)		168 (23.3)	12 (27.9)		53 (25.0)	12 (23.1)	
Obese (≥30.00)	111 (14.5)	93 (12.1)		95 (13.2)	16 (37.2)		36 (17.0)	15 (28.8)	
**Obstetrical characteristics**									
**Gravidity**									
Primigravida	286 (37.3)	262 (33.1)	0.085	272 (37.5)	14 (33.3)	0.586	80 (37.4)	17 (32.7)	0.528
Multigravida	481 (62.7)	529 (66.9)		453 (62.5)	28 (66.7)		134 (62.6)	35 (67.3)	
**Parity**									
Primipara	347 (49.8)	332 (45.5)	0.109	329 (50.2)	18 (42.9)	0.354	105 (53.6)	19 (39.6)	0.082
Multipara	350 (50.2)	397 (54.5)		326 (49.8)	24 (57.1)		91 (46.4)	29 (60.4)	
**Type of labour onset**									
Induced labour	191 (29.3)	184 (26.5)	0.248	171 (28.0)	20 (48.8)	0.005	55 (30.4)	19 (39.6)	0.226
Spontaneous labour	460 (70.7)	510 (73.5)		439 (72.0)	21 (51.2)		126 (69.6)	29 (60.4)	
**Mode of delivery** ^**6**^									
Spontaneous vaginal	563 (80.7)	450 (61.7)	<0.001	526 (80.2)	37 (88.1)	0.329	159 (81.1)	43 (89.6)	0.211
Assisted vaginal delivery	22 (3.2)	13 (1.8)		22 (3.4)	0 (0.0)		10 (5.1)	0 (0.0)	
Caesarean section	113 (16.2)	266 (36.5)		108 (16.5)	5 (11.9)		27 (13.8)	5 (10.4)	
**Obstetrical analgesia**									
Epidural/spinal	380 (54.4)	362 (49.7)	0.071	355 (54.1)	25 (59.5)	0.495	106 (54.1)	30 (62.5)	0.293
No analgesia	318 (45.6)	367 (50.3)		301 (45.9)	17 (40.5)		90 (45.9)	18 (37.5)	
**Preterm birth prevalence**									
GA<37 weeks (preterm)	37 (5.3)	56 (7.7)	0.069	32 (4.9)	5 (11.9)	0.064	5 (2.6)	3 (6.3)	0.193
GA≥ 37 weeks (term)	661 (94.7)	673 (92.3)		624 (95.1)	37 (88.1)		191 (97.4)	45 (93.8)	
**Gestational age (weeks**)	38.9 (1.7)	38.7 (2.0)	0.025	39.0 (1.6)	38.1 (2.9)	0.002	39.1 (1.4)	38.8 (2.5)	0.321
**Birth weight (grams**)	3378.6	3339.3	0.176	3385.3	3272.8	0.181	3358.6	3369.5	0.883
	(528.4)	(564.4)		(518.8)	(659.7)		(463.1)	(458.1)	

^1^Variables are presented as n (%), except for maternal age, gestational age at delivery and infant birth weight which are presented as mean and (SD)

^**2**^ Comparisons between women who did and who did not experience urinary incontinence at 12 months postpartum

^3^ Comparisons between women with mild and moderate/severe symptoms of urinary incontinence

^4^ Comparisons between women who reported mild and moderate/severe impairment of daily tasks due to urinary incontinence

^5^The education categories included: for High school category, some high school, graduated high school, or some post-secondary, and for Post-secondary category, graduated post-secondary, some graduate school, or completed graduate school

^6^Assisted vaginal deliveries included forceps, vacuum or both forceps and vacuum; Caesarean section included both emergency and elective caesarean sections

*p-values* from Chi square and Fisher’s exact tests

Percentages are calculated per column for each variable

Women who reported moderate to severe symptoms of UI were older (mean age 33.7 years, p = 0.004), likely to be obese (p<0.001), single (p = 0.043), less educated (p = 0.041) and more likely to have had labour induction (p = 0.005) than those women who reported mild symptoms of UI.

Among the 17% of women who reported any effect of UI on daily tasks (n = 267), 80.5% (n = 215) were mildly affected, and 19.5% (n = 52) reported moderate or severe impairment. Women who reported moderate to severe impairment of UI on maternal performance of daily tasks did not differ in their demographic and obstetrical characteristics or obstetrical events in labour and at delivery from the women who reported mild effects of UI on performance of daily tasks. There was an association between the frequency of UI symptoms and performance on daily tasks; women reporting moderate to severe UI also reported higher impairment on the performance of daily tasks (p<0.001).

In multivariable regression analyses, maternal age, pre-pregnancy BMI, and method of delivery were risk factors for experiencing moderate to severe impairment in performance of daily tasks due to UI at 12 months postpartum. Obese women had almost three times increased risk for developing moderate to severe impairment of maternal performance of daily tasks as compared to normal weight women (adjusted OR 2.84, CI 1.34, 6.02) (Table 3). Women who delivered vaginally, either spontaneously or with obstetrical assistance were at fourfold higher risk of moderate to severe impairment of maternal performance of daily tasks due to UI (adjusted OR 3.94, CI 1.52–10.23) in comparison to women who were delivered by C-section. In our model, co-existence of BP was not a predictive factor for impairment of maternal performance of daily tasks due to UI.

### Characteristics of women reporting both BP and UI

Almost 40% (n = 620) of women reported symptoms of both BP and UI at 12 months postpartum. They were more likely to be Caucasian (p = 0.023), obese (p = 0.018), had induced labour (p = 0.038) and delivered vaginally (p<0.001) compared to those women without BP and UI (S1 Table). No differences were found in the education, income, marital status, parity, maternal age, obstetrical analgesia or birth weight between women who reported both BP and UI and those who did not.

## Discussion

This study sought to determine the prevalence, severity, and the degree of impairment on maternal performance of daily tasks of BP, UI, and both BP and UI in healthy women at 12 months postpartum. Only few studies have examined the demographic and obstetrical factors related to the severity of functional impairment or quality of life in postpartum women. Comparison to other studies is limited to those reporting prevalence of these conditions several months postpartum. Previous studies report lower prevalence of UI in the late postpartum period, around 12% in a study by Burgio *et al* (2003) [33] and 24% by Woolhouse *et al* [26]. The findings from this study are similar to more recent reports on postpartum incontinence that also report higher UI prevalence at 12 months postpartum [34, 35]. In contrast to previous studies that assessed BP and UI either retrospectively or in distinct populations such as nulliparas, the present study analysed the prevalence and the characteristics of these symptoms in a pregnant population of primi- and multiparas drawn from a prospective, community-based pregnancy cohort representing a metropolitan population who received prenatal care in community clinics and obstetrical care for labour and at delivery in tertiary hospitals. In agreement with previous studies, our findings also support a high prevalence of BP during the postpartum period [28, 35]. Furthermore, the proportion of women affected by BP and the impairment of maternal performance of daily tasks due to BP has a higher impact on women’s health and performance of daily tasks at 12 months postpartum than that by UI. The key risk factors for functional impairment due to BP in our sample were obesity and parity and for UI, risk factors included maternal age, obesity and vaginal delivery.

In multivariate analyses, higher maternal age remained significant for the development of moderate to severe impairment of daily tasks due to UI (OR 1.1, 95% 1.03–1.18) after controlling for demographic and obstetrical variables. In a systematic review of the literature, Hijaz *et al* (2012) identified five short term prospective studies of postpartum UI that reported a relative risk for advanced maternal age ranging from RR = 1.1 to 1.5. This suggests that the development of UI postpartum increases with increased maternal age [36]. However, other studies have shown only a marginal association [37] or no association between maternal age and UI at 1 year postpartum.

Rortveit *et al* [11] reported the prevalence of any incontinence to be 10.1% in nulliparous women, 15.9% in women who had delivered by C-section and 21% in women delivering vaginally. Three later studies presented results supporting increased risk of UI with vaginal delivery and implied that C-section may be protective of UI [1, 14, 15]. In a population-based random sample of 3,205 black, Hispanic, and white women aged 30–79 years, Connolly *et al* [16], reported that those women who had a history of least one vaginal delivery experienced a two fold increase in risk of moderate to severe symptoms of UI compared to women who had never been pregnant (p = 0.032) or women who delivered by C-section (p = 0.002). Reasons women give for requesting C-section for first time delivery include the fear of perineal damage and UI [38, 39]. Some obstetricians are supportive of offering C-section to decrease risk of pelvic floor injury and incontinence related to vaginal delivery [40]. Some evidence supports higher rates of pelvic floor damage and UI rates among women delivering vaginally but suggests the risk of UI is higher among women who have had C-sections than among nulliparous women [11]. We found in our study a 4 fold risk of moderate to severe UI in women delivering vaginally compared with women who delivered by C-section (both elective and emergency). This implies that the protective effect of C-sections occurs regardless of the time during parturition when is offered. However the protective effect of C-section may decrease over time given aging or subsequent deliveries [41]. Furthermore, the risks and short- and long-term negative impact of C-section should not be dismissed. Other UI predictors included parity, obesity, maternal smoking and infant birth weight >4000 g [17].

Cheng and Li (2008) noted that if women consider incontinence as a normal occurrence of pregnancy and delivery, they may not seek help from their health care provider and contribute to underreporting. The AOB questionnaires increased awareness of study participants to UI symptomatology and gave women an opportunity to self-report UI.

Several factors have been proposed associating BP during pregnancy and the postpartum period including demographic factors (age and occupation), parity, BMI and weight gain during pregnancy, but overall the results are inconsistent and inconclusive. Many studies found a high prevalence of BP in women after childbirth [42, 43] and several reported an increase in BP over time, from 5 to 12 months postpartum [21]. In a longitudinal cohort, Saurel-Cubizolles *et al* (2000) reported that the prevalence of BP increased significantly from 47.4% at 5 months to 64.7% at 12 months for women in France but only slightly, from 49.4% at 5 months to 50.9% at 12 months, for women in Italy. Other studies report a much lower prevalence of BP at one year postpartum [24, 43]. For example, Schytt *et al* [43] found 33.7% prevalence at 12 months in Swedish women, albeit higher than at 2 months after childbirth (28%). In a large Australian study, BP affected more than half of the sample in the first 8 weeks (53%), with the rates declining slightly over the first 6 months postpartum (45%) [24]. However, most of these studies present only crude estimates of the association between BP and childbirth and very few addressed the risk factors that may accompany these associations. We have explored these associations and assessed the influence of the demographic and obstetrical factors on the severity of the symptoms and impairment of daily activities. The associations with specific obstetrical interventions, birth weight and maternal weight gain during pregnancy warrant further investigation.

Very little is known about the impact of these two symptoms on the maternal performance of daily tasks in the postpartum period. Several studies assessed functional impairment and quality of life due to BP or UI during pregnancy and postpartum. Pregnant women with BP report higher scores on disability index and impact on occupation, ability to perform jobs around the house, hobbies and social life than women without BP [44]. Postnatally, more women who experience UI report impact on their lives compared than antenatally [45].

Our study suggests that although BP and UI are common occurrences after childbirth, for most women the symptoms are mild and do not impair performance of daily chores, being these related or not to the childrearing. However, women who do report impaired functionality may undergo lifestyle changes with impact on general health [45]. To improve the level of functionality and further the effects on quality of life for mothers and their families, health care professionals may inform pregnant women about potential UI and recurrent BP and treatment options following pregnancy [46, 47].

Several limitations of our study should be considered. The symptoms of UI and BP were self-reported, and were not confirmed by clinical evaluation. In addition, we assessed the severity of UI based on frequency of symptoms rather than amount of leakage. Severity of BP was also based on frequency of symptoms. Several studies have shown that self-reported symptoms of BP and UI are valid and consistent when assessing either current symptomatology and/or changes over time [48]. We have shown a direct positive relationship between UI and BP on the impairment of daily activities in mothers using frequency of symptoms as a proxy for severity of either UI and BP. Women were not asked about symptoms of UI and/or BP prior to this pregnancy and delivery, and, therefore, we were unable to assess the presence of UI or BP before and/or during pregnancy. However, if the participants may have previously suffered of these conditions, it is likely that this was, at least partially, covered in the questions related to severity of symptoms as both conditions may have been exacerbated by the current delivery. Some evidence indicates only a small change in the prevalence of UI in both multiparous and primiparous women from pregnancy (42%) to postpartum (38%) symptoms [49], the onset of UI during pregnancy being reported as an independent risk factor for persistence of UI during postpartum period [13, 50]. In addition, obese women may have had UI prior to pregnancy as increased body weight is known to contribute to chronic damage to pelvic floor musculature [17], information that was not available to this study. A recent study from Gutke *et al* suggests that the impact of back pain on postpartum disability was equivalent irrespective of symptoms in lumbar or pelvic areas [51].

The information collected from the participants did not allow differentiating the symptoms of stress from urge incontinence and mixed type incontinence or if these subtypes of UI have different impact on performance of daily tasks. Based on the published evidence and considering the socio-demographic and clinical factors we can only assume that the majority of these women may be suffering from urge incontinence. Further research is warranted to elucidate the influence of a specific type of UI on daily tasks and the degree on impairment in postpartum women. We were not able to assess the impact on daily activities of women who had both UI and BP. These women were assessed only within the UI and BP categories; we were unable to assess if presence of both symptoms had additive effects on their ability to perform daily chores.

BP was not specifically defined in our study. We expect that women experiencing any BP, located in the upper, lower or pelvic girdle regions would have been reported. BP of any sort compromising daily tasks is a concern for postpartum women.

Major strengths of this study include: The sample was large, drawn from a prospective pregnancy cohort, for which the participants were selected from the general population, thus, reducing the selection bias. Furthermore, the demographic characteristics of the AOB Study participants are reflective of the pregnancy population in urban centres Canada, which suggests that this sample is representative in terms of demographics and key obstetrics characteristics and our results are generalizable. Our estimates showed that BP and UI occurred within a wide cross section of women in AOB, consistent with other longitudinal cohorts [34, 50].

Additionally, women from our study were considered low obstetrical risk, received prenatal care in community centers and delivered in tertiary hospitals. We were able to assess the outcomes from all modes of delivery, including assisted deliveries, emergency and elective C-section. We also assessed the role of induction of labour in the occurrence and severity of these symptoms, and controlled for gestational age, body mass and parity, variables that could have influenced the outcomes of the pregnancy.

## Conclusion

Although physical problems such as BP and UI are commonly associated with the postpartum period and often regarded as transient, they are strongly related to impairment of maternal performance of daily tasks. Raising awareness of health professionals about these debilitating conditions may change attitudes towards these patients to receive the care they need. Demographic and obstetrical factors such as age, parity, BMI and mode of delivery should be considered in the evaluation of women to identify those at risk and suggest preventative measures. In addition, appropriate counselling of pregnant women about potential UI and recurrent BP during pregnancy and postpartum and treatment options following pregnancy may contribute to improved functioning and performance of daily tasks, reflecting in better quality of life for new mothers and their families.

## Supporting Information

S1 Table Demographic and obstetrical characteristics of women reporting symptoms of both back pain and urinary incontinence at 12 months postpartum. (DOCX)
