# Supplementary material for: The Influence of Back Pain and Urinary Incontinence on Daily Tasks of Mothers at 12 Months Postpartum
Source: PLoS One. 2015 Jun 17;10(6):e0129615. doi: 10.1371/journal.pone.0129615 (PMC4471341; doi:10.1371/journal.pone.0129615)
Supplement: S1 Table — (DOCX) [file pone.0129615.s001.docx]

**S1 Table. Demographic and obstetrical characteristics women reporting symptoms of both back pain and urinary incontinence at 12 months postpartum**

| **Variables^1^** | **Back pain and urinary incontinence** | **No back pain and urinary incontinence or only back pain or urinary incontinence** | **p-values^2^** |
| --- | --- | --- | --- |
|  | *620 (39.4%)* | *954 (60.6%)* |  |
|  |  |  |  |
| **Demographics** |  |  |  |
|  |  |  |  |
| **Mean maternal age (years)** | 31.5 (4.5) | 31.4 (4.3) | 0.242 |
| **Ethnicity** |  |  |  |
| White/Caucasian | 527 (85.1) | 764 (80.7) | 0.023 |
| Other | 92 (14.9) | 183 (19.3) |  |
| **Household income** |  |  |  |
| < $80,000 | 167 (27.7) | 263 (28.9) | 0.634 |
| ≥ $80,000 | 435 (72.3) | 648 (71.1) |  |
| **Education**^3^ |  |  |  |
| High school | 151 (24.4) | 209 (22.1) | 0.290 |
| Post-secondary | 468 (75.6) | 737 (77.9) |  |
| **Marital Status** |  |  |  |
| Married/common law | 587 (94.8) | 910 (96.1) | 0.234 |
| Single | 32 (5.2) | 37 (3.9) |  |
| **Pre-pregnancy body mass index** |  |  |  |
| **(BMI, kg/m²)** |  |  |  |
| Underweight (< 18.50) | 17 (2.8) | 37 (4.0) |  |
| Normal weight (18.50-24.99) | 356 (58.2) | 579 (62.7) | 0.018 |
| Overweight (25.00-29.99) | 139 (22.7) | 204 (22.1) |  |
| Obese (≥30.00) | 100 (16.3) | 104 (11.3) |  |
|  |  |  |  |
| **Obstetrical characteristics** |  |  |  |
|  |  |  |  |
| **Gravidity** |  |  |  |
| Primigravida | 226 (36.7) | 322 (34.1) | 0.293 |
| Multigravida | 389 (63.3) | 621 (65.9) |  |
| **Parity** |  |  |  |
| Primipara | 279 (49.7) | 400 (46.2) | 0.197 |
| Multipara | 282 (50.3) | 465 (53.8) |  |
| **Type of Labour** |  |  |  |
| Induction of labour | 163 (31.0) | 212 (25.9) | 0.038 |
| Spontaneous onset | 362 (69.0) | 608 (74.1) |  |
| **Type of delivery**^4^ |  |  |  |
| Spontaneous vaginal | 451 (80.2) | 562 (65.0) | <0.001 |
| Assisted vaginal delivery | 14 (2.5) | 21 (2.4) |  |
| Caesarean section | 97 (17.3) | 282 (32.6) |  |
| **Obstetrical analgesia** |  |  |  |
| Epidural/spinal | 300 (53.4) | 442 (51.1) | 0.399 |
| No analgesia | 262 (46.6) | 423 (48.9) |  |
| **Preterm births prevalence** |  |  |  |
| GA < 37 weeks | 28 (5.0) | 65 (7.5) | 0.058 |
| GA ≥ 37 weeks | 534 (95.0) | 800 (92.5) |  |
| **Gestational age (weeks)** | 38.9 (1.7) | 38.8 (1.9) | 0.042 |
| **Birth weight** (grams) | 3382.2 (529.0) | 3343.2 (558.6) | 0.39 |

^1^Variable are presented as n (%), except for maternal age, gestational age at delivery and birth weight which are presented as mean (SD)

^2^For each variable the comparisons were made between women who experienced both back pain and urinary incontinence at 12 months postpartum and women who did not experience back pain or urinary incontinence or reported symptoms of either back pain or urinary incontinence

^3^The education classification categories were High school category, which included some high school, graduated high school, or some post-secondary; and Post-secondary category, which included graduated post-secondary, some graduate school, or completed graduate school

^4^Assisted vaginal deliveries included forceps, vacuum or both forceps and vacuum; Caesarean section included both emergency and elective caesarean sections

Percentages are calculated per column for each variable
